# Analyzing the Bolometric Performance of Vanadium Oxide Thin Films Modified by Carbon Nanotube Dispersions

**DOI:** 10.3390/ma16041534

**Published:** 2023-02-12

**Authors:** Usha Philipose, Chris Littler, Yan Jiang, Alia Naciri, Michael Harcrow, A. J. Syllaios

**Affiliations:** Department of Physics, University of North Texas, Denton, TX 76203, USA

**Keywords:** vanadium oxide, carbon nanotubes, noise, dispersion density, temperature coefficient of resistance, bolometers

## Abstract

The influence of carbon nanotube (CNT) dispersions on the electrical properties and noise signal amplitude of $VO_{x}$ films is investigated. For a critical range of the CNT dispersion density on $VO_{x}$ films, the intrinsic properties of the $VO_{x}$ films are modified by the CNTs. The CNT concentrations reported in this work are about 0.3 μg/cm${}^{2}$ and 1.6 μg/cm${}^{2}$, allowing for low density and high density dispersions on the $VO_{x}$ film surface to be investigated. These values are higher than the percolation threshold of about 0.12 μg/cm${}^{2}$ for these films. The composite film exhibits a significant reduction in the temperature coefficient of resistance (TCR) (from ≈3.8% $K^{-1}$ to ≈0.3% $K^{-1}$) for high density dispersions. In contrast, while $VO_{x}$–CNT composites with low density single wall CNT dispersions exhibit no significant change in TCR values, an approximate two orders of magnitude reduction in the low frequency 1/f noise is measured. The noise signal amplitude measured at 0.1 V and at 1.0 Hz reduces from 6 × $10^{-5}$$V/\sqrt{(}Hz)$ for $VO_{x}$ films to 5 × $10^{-7}$$V/\sqrt{(}Hz)$ for the low density SWCNT dispersion on $VO_{x}$ film and to 3 × $10^{-6}$$V/\sqrt{(}Hz)$ for the low density MWCNT dispersion on $VO_{x}$ film. The CNT concentration is the critical factor for yielding the observed changes in conductivity and low frequency noise. The results presented in this work provide a better understanding of $VO_{x}$-based composites, thereby enabling the development of new, versatile and functional materials for device applications.

## 1. Introduction

Improving infrared (IR) imaging technology through engineering the properties of the temperature-sensing membrane is fundamental to the development of the next generation of microbolometers [1,2]. This technology is essential to applications ranging from defense and civilian and military security to astronomy and medical research. A microbolometer is composed of a thermally isolated thin film of a material (membrane) whose variation in resistance with temperature is monitored and correlated with the scene temperature. When microbolometers are arranged in a two-dimensional array of pixels and placed at the focal point of imaging optics, i.e., a focal plane array (FPA), a thermal image of a scene is produced. The material of choice for current infrared (IR) FPAs is vanadium oxide ($VO_{x}$) [3,4,5,6]. In some films, the two-dimensional (2D) layered structure of $VO_{x}$ has mixed oxidation states of vanadium (V) and exhibits a desirable resistance (R), a high temperature coefficient of resistance (TCR) and low 1/f noise. In a previous work by Sood et al. [7], the integration of CNTs into $VO_{x}$ films was studied, where the focus was to improve the TCR for low noise and the high dynamic range microbolometer focal plane array performance.

In this work, we present a detailed study of the electrical performance and noise characteristics of $VO_{x}$-based composite films formed by dispersing varying densities of carbon nanotubes (CNTs) on the film surface. Devices were fabricated by dispersing both single-walled CNTs (SWCNT) and multi-walled CNTs (MWCNT) on $VO_{x}$ films. The unique one-dimensional (1D) properties of the CNTs, along with their promising electrical, optical and mechanical properties, make them ideal candidates for many applications, including field emission devices and sensors. The promise of CNTs in uncooled bolometer applications was demonstrated by Itkis et al. [8], who showed that suspended CNT films annealed at 670 K exhibit TCR values ranging from −1% to −2.5% $K^{-1}$, making CNTs promising bolometric materials. However, due to their metallic nature, CNT arrays have very low resistance, which is detrimental to their use in bolometers, since low resistance values limit the signal output of bolometers. To circumvent this challenge, several groups have reported on polymer-–CNT composites as bolometric materials. The major drawback of such composites is the poor electrical and thermal conductivities of the polymers. In view of this, Q. He et al. [9] have studied the physical and chemical structure as well as the optical and electrical properties of $VO_{x}$–CNT composite films with different CNT concentrations. Their work showed that at critical CNT concentrations (≈4%), the chemical interactions between the $VO_{x}$ film and the SWCNTs result in an enhancement of the $VO_{x}$ film’s optical and electrical properties as well as preserving high film uniformity. Outside of this limit, the authors claimed that the optical and electrical properties and film uniformity is negatively impacted. In this work, we present a detailed study of the temperature-dependent electrical properties and noise characteristics of $VO_{x}$–CNT composite films. We show that these properties are either significantly degraded or enhanced depending on the CNT concentration.

The key features of uncooled bolometers are: (i) a higher change in resistance for small changes in temperature (high TCR), (ii) a reduced electrical noise level and (iii) increased IR absorption [10,11,12]. The figure of merit of the microbolometer is the Noise Equivalent Temperature Difference (NETD), effectively a measure of the temperature difference that a pixel array can differentiate. It is quantitatively defined by Equation (1) [13]:

(1) $$NETD=F_{system\;optics}\frac{V_{noise}}{A_{pixel}R(\Delta P/\Delta \left(T_{Blackbody}\right)}$$

where $F_{system\;optics}$ is related to the optical configuration of the detector, $V_{noise}$ is the electrical noise associated with the detector, R is the responsivity, $A_{pixel}$ is the pixel area and ΔP/ΔT defines the change in power radiated by a blackbody per unit change in its temperature. A simplified form of this equation is:

(2) $$NETD\equiv device\;parameters\frac{V_{noise}}{(TCR)\cdot \eta }$$

As indicated in Equation (2), the NETD is directly proportional to the noise signal ($V_{noise}$) and inversely proportional to the TCR and absorption constant (η). A $VO_{x}$–CNT composite film is of interest due to the high η of CNTs in the IR range and $VO_{x}$ being the current industry standard for thermal imagers [14,15,16].

In microbolometer technology, the conductivity of the sensing material ($VO_{x}$) can generally be described by hopping transport, expressed by Equation [17]:

(3) $$\sigma \left(T\right)=\sigma_{o}\;exp\left[-{\left(\frac{T_{o}}{T}\right)}^{p}\right]$$

where $\sigma_{o}$ is the conductivity pre-factor, $T_{o}$ is the characteristic temperature, providing a measure of the degree of disorder in the film, and *p* is the hopping exponent whose value depends on the mode of hopping. Mott [18,19] considered a constant density of states and neglected Coulomb interactions to show that the hopping exponent has a value of *p* = ¼. However, Efros and Shklovskii (ES) [20,21] later pointed out that, at low temperatures, the density of states near the Fermi level is not constant. As an electron hops from one site to another, it leaves behind a hole, and the system uses energy to overcome this electron–hole coulomb interaction. This results in a linear decrease in the density of states with energy. This vanishing density of states, called the Coulomb gap, results in a temperature dependence of the conductivity that can still be described by Equation (3), but with *p* = ½. The difference between the Mott and ES-VRH conductivity is in the details of their localization parameters, the density of states at the Fermi level and the interactions between states, all of which influence the temperature dependence of the conductivity. There are reports of both Mott and ES variable range hopping conduction observed in $VO_{x}$ films [22,23,24]. In most cases, the hopping conduction mechanism is determined by analyzing the semi-logarithmic plot of resistance (R) versus either $T^{-1/4}$ (Mott-VRH), $T^{-1/2}$ (ES-VRH) or $T^{-1}$ (Near Neighbor Hopping—NNH). However, there are cases where the data can be fitted equally with either $T^{-1/4}$, $T^{-1/2}$ or $T^{-1}$, making it difficult to identify the conduction mechanism. In order to avoid this ambiguity, the hopping exponent, *p*, can be determined using a Resistance Curve Derivative Analysis (RCDA) technique [25]. In this method, the logarithmic conductivity data with temperature is numerically differentiated, and the quantity “*w*” is determined using the expression:

(4) $$w=\frac{d(log\sigma )}{d(logT)}=p{\left(\frac{T_{o}}{T}\right)}^{p}⟹log\;w=-p\;logT+log\;p{\left(T_{0}\right)}^{p}$$

To validate a given conduction mechanism, the exponent *p* is obtained from the slope of a log *w* versus log *T* plot.

The temperature coefficient of resistance (TCR) is another parameter that can be used to validate the conductivity regime. It is defined by Equation [17]:

(5) $$\left|TCR\right|=|\frac{1}{\sigma }\;\frac{d\sigma }{dT}|=p\;\frac{T_{o}^{p}}{T^{p+1}}$$

For each hopping mechanism described above, the TCR has a $T^{\left(p+1\right)}$ power law dependence, controlled by the characteristic temperature, $T_{o}$. For example, in the Mott regime, TCR is proportional to $T_{o}^{\left(1/4\right)}$. The TCR data is obtained by a numerical differentiation of the resistance or conductivity versus temperature data. Both RCDA and TCR versus temperature are sensitive derivative techniques, allowing the extraction of p and thus the conductivity mechanism to be determined.

## 2. Materials and Methods

The $VO_{x}$ films used in this study (obtained from Magnolia Optical Technologies, Woburn, MA, USA ) were deposited by a DC sputtering technique on a $Si/SiO_{2}$ substrate. The thickness of the $SiO_{2}$ was 280 nm. The 220 nm-thick $VO_{x}$ films had an [O]/[V] ratio of about 1.8. The CNTs used in this work, both MWCNTs and SWCNTs, were purchased from Nano-Integris (0.1 g/100 mL) and had diameters in the range of 30–40 nm and 10–15 nm, respectively. A drop of the parent CNT solution was diluted 25:1 or 100:1 with isopropyl alcohol to create high and low packing density devices. The vials containing the dilute solutions were vigorously shaken to ensure efficient de-bundling. The surfactant shell of sodium dodecyl-sulfate (SDS) on the CNTs was removed following a procedure described in our earlier work [26]. This was done prior to device fabrication to to ensure a good interface between the CNTs and the metal contacts. The CNT concentrations were varied from 0.3 μg/cm${}^{2}$ to about 1.6 μg/cm${}^{2}$ to allow for low-density and high-density dispersions on the $VO_{x}$ film surface. The dispersion was achieved using a meniscus dragging dispersion (MDD) technique [26,27]. Metal contacts (100 nm Au contacts) to the $VO_{x}$/CNT composite were made by photolithography using an MJB3 mask aligner, followed by thermal evaporation of the metal contacts. Electrical measurements were made under high vacuum ($10^{-5}$ to $10^{-6}$ Torr) in a cold finger cryostat. The device was attached to a 24-pin ceramic holder mounted into a socket and maintained in physical contact with the copper cold finger in a Janis cryostat. The device temperature was controlled using a Lakeshore 332 Temperature controller and regulated by a slow liquid nitrogen flow. The electrical conductivity and TCR measurements were made using an Agilent Semiconductor Device Analyzer (B1500A), with the device biased from 0 to 1.0 V. The data were collected at 5 K intervals over a temperature range from 250 K to about 390 K. The Noise measurements were done at room temperature under dark conditions, within an electrically insulated chamber to minimize outside disturbances. A Keithley 428 source was used to bias the device and also functioned as a low noise current amplifier. The gain for each device was adjusted by a variable feedback resistor within the pre-amplifier circuit. The amplified signal was converted to a noise voltage–frequency spectrum by an HP 35670A dynamic signal analyzer (DSA). X-ray diffraction (XRD) measurements were performed on a 1.0 cm${}^{2}$ sample that was cleaved from the $VO_{x}$ wafer. The measurement was performed using a Rigaku Ultima III XRD diffractometer ($CuK_{\alpha }$, step size 0.02°) after appropriate calibration of the system.

## 3. Results and Discussion

**Figure 1:**
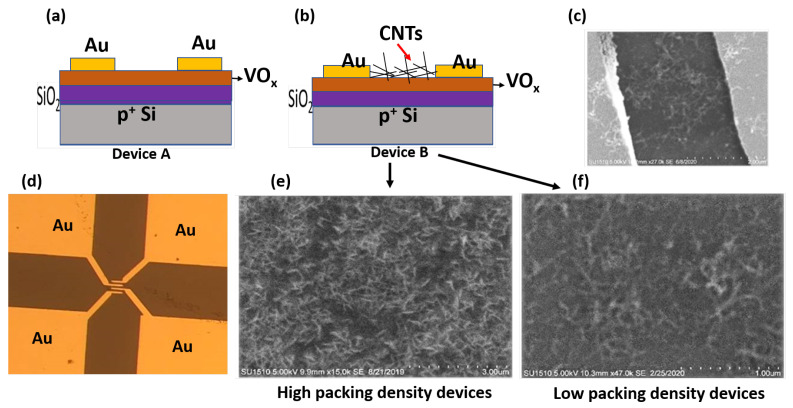
(**a**) Schematic of device fabricated on a $VO_{x}$ film; (**b**) schematic of device fabricated on a $VO_{x}$/CNT composite system; (**c**) SEM image of Au contacts on the $VO_{x}$/CNT composite system; (**d**) optical image of device showing four gold contacts on a VO${}_{x}$ film; (**e**) SEM image of the $VO_{x}$/CNT composite system with a high density of CNT dispersions (≈1.6 μg/cm${}^{2}$); (**f**) SEM image of the $VO_{x}$/CNT composite system with a low density of CNT dispersions (≈0.3 μg/cm${}^{2}$).

To facilitate a systematic study of the electrical and noise characteristics of $VO_{x}$ films and of $VO_{x}$/CNT composite systems, two basic types of devices were fabricated, as shown in Figure 1a,b. Device A (Figure 1a), $VO_{x}$ film with no CNT dispersions, allowed for a study of the electrical conductivity and noise signal of the $VO_{x}$ film. The CNT concentrations in device B (Figure 1b) were varied to study the effect of CNT density (high packing density device (Figure 1e) and low packing density device (Figure 1f)) on the device performance in terms of its effect on both electrical conductivity and noise signal.

The estimation of the CNT packing density and determination of the percolation threshold has been detailed in one of our earlier works [26]. Figure 1c is an SEM image of the low packing density device contacted by two Au electrodes, while Figure 1d is an optical image showing the configuration of the metal pads in the fabricated device.

### 3.1. Determination of Conduction Mechanism and TCR of Device A: $VO_{x}$ Films

**Figure 2:**
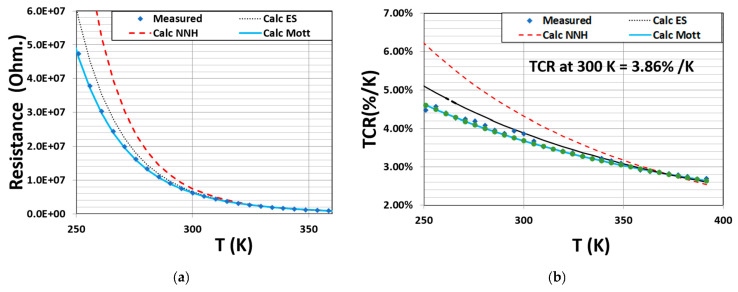
(**a**) Resistance–temperature measurement data of the $VO_{x}$ film with blue discrete points representing measured data. The red (dashed curve), black (dotted curve) and blue (solid curve) represent fits corresponding to NNH, ES and Mott, respectively; (**b**) TCR measurement data and the fit to the conduction models for device A comprising of the $VO_{x}$ film.

Figure 2 shows the resistance and TCR values as a function of temperature for device A ($VO_{x}$ film). The results were analyzed to determine which of the hopping conduction mechanisms is dominant.

The discrete points in the plots represent the measured data. Figure 2a,b shows that the R–T and TCR–T data are well described by Mott VRH conduction. The TCR value of these films measured at room temperature was about 3.86% $K^{-1}$.

**Figure 3:**
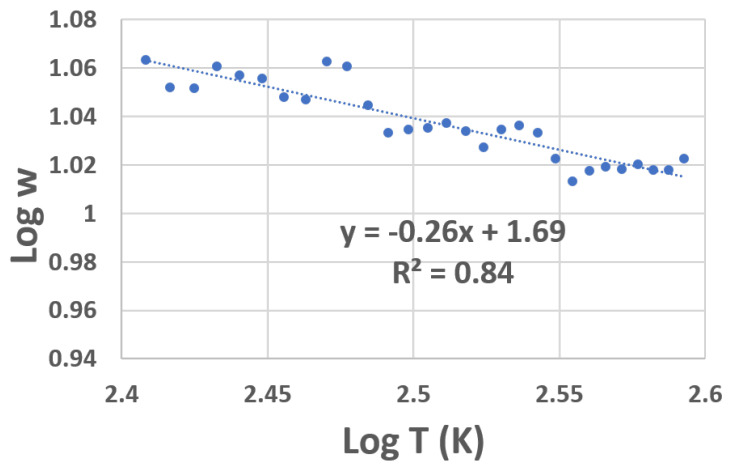
RCDA of device A ($VO_{x}$ film) showing variation in log *w* with log *T*. The slope of the best line fit is 0.26, which indicates Mott VRH conductivity.

Figure 3 shows the results of the RCDA analysis for the resistance data obtained for device A (plotted in Figure 3). The line of best fit to the data (shown as a dotted line) gives the exponent a value of ≈0.25, indicating that the conduction mechanism of the $VO_{x}$ film under study in the given temperature range follows Mott VRH.

**Figure 4:**
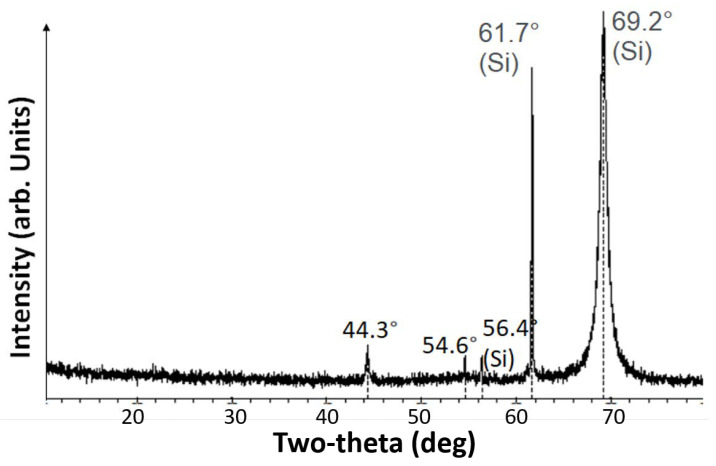
X-ray diffraction spectrum obtained from the $VO_{x}$/$SiO_{2}$/Si sample, showing peaks corresponding to $VO_{2}$, $V_{2}O_{5}$ and $V_{4}O_{9}$ crystalline phases.

The accuracy of the linear fit, as estimated by the $R^{2}$ value, is about 84 %. Most $VO_{x}$ films contain polycrystals distributed in an amorphous matrix comprised of $VO_{x}$ with different oxidation levels [24]. The conduction mechanism in these films depends on the film growth conditions, which would determine its density of states, and the distribution of localized states around the Fermi level. In a highly disordered film, a Coulomb gap exists at all temperatures and the conduction would most likely follow ES-VRH. On the other hand, in less disordered films, the carriers have enough energy to overcome the Coulomb gap, resulting in a fairly constant density of states. In such systems, conduction is governed by Mott VRH. The presence of localized states and their energy distribution, as determined by the concentration of the [V]:[O] defects (dependent on the processing conditions) is the most likely cause for Mott conduction in these films. The $VO_{x}$ films under study in this paper were characterized by X-ray diffraction (XRD) measurements (Figure 4).

The peaks at 56.4${}^{\circ }$, 61.7${}^{\circ }$ and 69.2${}^{\circ }$ are all associated with $SiO_{2}/Si$. The two additional peaks measured at 44.33${}^{\circ }$ and at 54.63${}^{\circ }$ were analyzed based on the library of XRD peaks on Jade pdf card ($VO_{2}$, PDF # 00-009-0142, 00-019-1401, 00-033-1441; $V_{2}O_{5}$ PDF# 00-009-0387, 00-041-1426). The peak at 44.3${}^{\circ }$ is attributed to $VO_{2}$ and also to $V_{2}O_{5}$. The peak at 54.6${}^{\circ }$ is attributed to $VO_{2}$ and $V_{4}O_{9}$. These results show that crystalline phases corresponding to $VO_{2}$, $V_{2}O_{5}$ and $V_{4}O_{9}$ are present in the amorphous $VO_{x}$ film matrix and is the most likely cause for the Mott conduction in these films.

### 3.2. Determination of Conduction Mechanism and TCR of Device B: $VO_{x}$/CNT Composite Films

#### 3.2.1. Analysis of the Effect of SWCNT Dispersions

**Figure 5:**
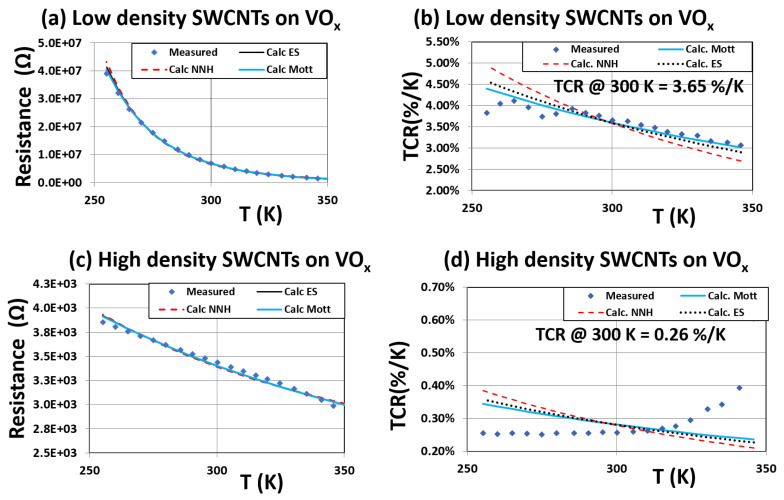
Resistance –temperature and TCR data for low density of SWCNTs on $VO_{x}$ films (**a**,**b**) and high packing density of SWCNTs on $VO_{x}$ films (**c**,**d**).

To study the effect of CNT dispersion on the conduction of the $VO_{x}$ films, two devices were fabricated. They were made from low (≈0.3 μg/cm${}^{2}$) and high (≈1.6 μg/cm${}^{2}$) packing density SWCNT dispersions on $VO_{x}$ films. The percolation threshold for the CNT network was estimated to be in the range of 0.10 μg/cm${}^{2}$–0.14 μg/cm${}^{2}$ for SWCNT and MWCNT dispersions. The devices under study in this work have dispersion densities higher than the percolation threshold. Figure 5 shows the R–T and TCR data for the two sets of devices. Figure 5a,b corresponds to a low density of CNTs on the $VO_{x}$ film surface, while Figure 5c,d corresponds to a high SWCNT dispersion density. Figure 5a is the R–T data obtained for device B with a low-density of SWCNT dispersion on the $VO_{x}$ film. Though the R versus T dependence continues to follow a similar trend as that observed for $VO_{x}$ films, the conduction mechanism is hard to identify since the data appear to fit all three trend lines corresponding to Mott, Efros and NNH. The fit to any one mechanism was not as clear as that obtained for the $VO_{x}$ film (shown in Figure 2a).

**Figure 6:**
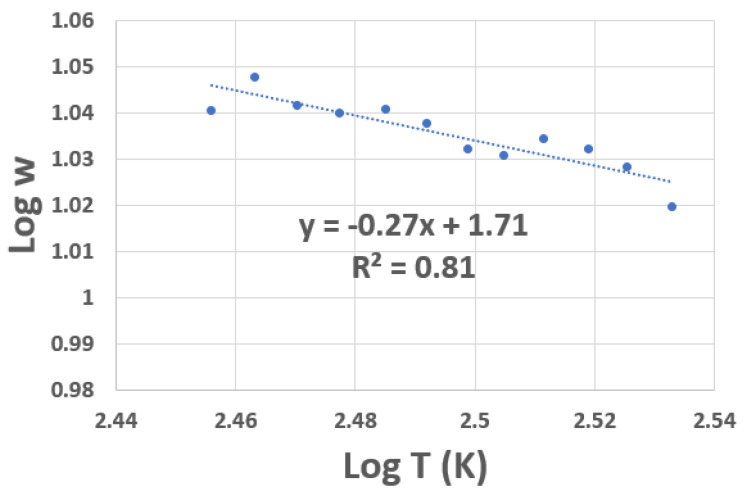
RCDA of device B (low packing density SWCNT on $VO_{x}$) showing variation in log *w* with log *T*. The slope of the best line fit is 0.27, which indicates Mott VRH conductivity.

An RCDA analaysis of the R–T data was performed on the low-packing density SWCNT composite device and the results are shown in Figure 6. The value of *p* is ≈0.25, which again confirms Mott VRH conduction. The variation in TCR with T for this device is shown in Figure 5b; the TCR value at room temperature being about 3.65% $K^{-1}$. In the high temperature range, the TCR data for this device follows the TCR data for the $VO_{x}$ film (Figure 2a), especially above room temperature. There is a significant shift in the TCR data in the low temperature regime.

These results reveal that after dispersion of a low-packing density of SWCNTs on the $VO_{x}$ film, there is no significant change in the electrical conductivity of this composite system, but there is a small reduction in the TCR value of this device compared to that of the $VO_{x}$ film (from 3.86% $K^{-1}$ to 3.65% $K^{-1}$). The conduction mechanism continues to be in the Mott regime.

The SWCNT concentration on the $VO_{x}$ film was increased to study the device characteristics for a high SWCNT packing density distribution. Figure 5c,d shows the R–T and TCR data for the high packing density device. As seen in Figure 5c,d, there is a significant decrease in both the resistance and TCR of these films. The R–T data does not exhibit a clear trend to any of the known conduction mechanisms. There is an almost four orders of magnitude increase in the film conductivity and an order of magnitude decrease in TCR (from 3.86% $K^{-1}$ to 0.26% $K^{-1}$).

These results agree well with the findings reported by He et al. [9], who reported a modification of the chemical structure and physical properties of $VO_{x}$ films by SWCNTs. In that work, the authors showed a significant change in the crystallinity, composition and morphology of the $VO_{x}$ film following SWCNT dispersion. The addition of SWCNTs resulted in strong chemical interactions between the $VO_{x}$ film and the SWCNTs. These interactions saturated at some critical CNT concentration on the $VO_{x}$ film surface and were reported to be influenced by the the [V]:[O] concentration, which resulted in changes in the energy band gap, optical absorption, room temperature conductivity and TCR of the composite film. In this reference [9], the authors presented data to show that at low SWCNT concentrations (less than 4 wt.%), the $VO_{x}$/CNT film shows poor electrical conductivity, while for high SWCNT concentrations (greater than 6 wt.%), the composite film exhibits a low TCR and poor morphology uniformity. The R–T and TCR data for device B under study in this paper agree well with the findings of this published work. Our results indicate that the R–T and TCR results for the low packing density composite was not significantly influenced by the addition of CNTs to the $VO_{x}$ film, contrary to the high packing density composites, where an increased conductivity and significantly reduced TCR was measured. As reported by He et al. [9], at high enough SWCNT concentrations, a chemical interaction occurs between the $VO_{x}$ and the SWCNTs through the oxygen atoms, resulting in the oxidation of SWCNTs after composite formation. Such chemical interactions facilitate electron transfer from 1-D SWCNTs to 2D $VO_{x}$ through oxidation and reduction reactions, resulting in an increase in the oxygen vacancies in the $VO_{x}$ film and enhancing its electrical conductivity. Moreover, at high CNT concentrations, there exists multiple conductive paths to facilitate electron transfer (increased conductivity), whereas low density CNT dispersions modify the $VO_{x}$ film surface, as evidenced by small changes in conductivity and TCR values.

#### 3.2.2. Analysis of the Effect of MWCNT Dispersions

**Figure 7:**
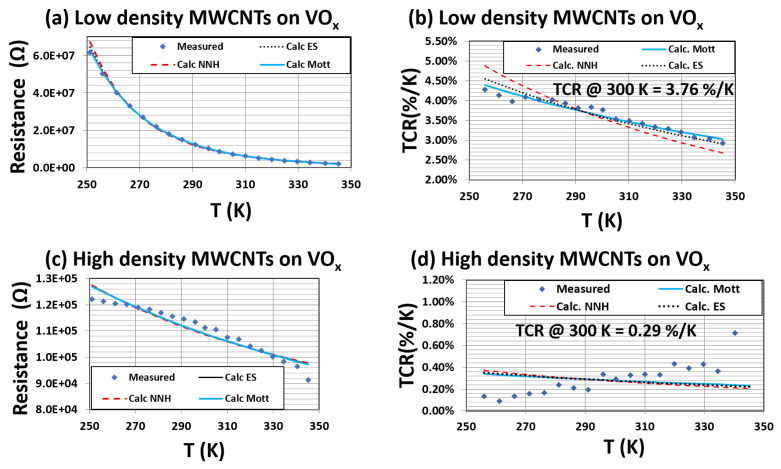
Resistance–Temperature and TCR data for a low density of MWCNTs on $VO_{x}$ films (**a**,**b**) and a high packing density of MWCNTs on $VO_{x}$ films (**c**,**d**).

For the MWCNT dispersion, the low packing density dispersion of MWCNTs on $VO_{x}$ resulted in a minimal change to the conductivity of the composite (Figure 7a) and a very small change (from 3.86% $K^{-1}$ to 3.76% $K^{-1}$) in the TCR values (Figure 7b). However, for high packing density dispersions, the increased conductivity values (by about two orders of magnitude as shown in (Figure 7c)) and reduced TCR (from 3.86% $K^{-1}$ to 0.26% $K^{-1}$) (Figure 7d) showed similar trends to what was observed for SWCNT dispersions.

These results validate the fact that the low density dispersion of MWCNTs does not significantly impact the conductivity of the $VO_{x}$ films. However, it does modify the film surface, as evidenced by the change in the R–T and TCR–T measurements. RCDA analysis of the MWCNT conductivity showed that the conduction did not follow any of the known hopping conduction mechanisms.

### 3.3. Noise Measurements

A major factor limiting the performance of current uncooled microbolometers is noise in the bolometer detectors, which occurs due to fluctuations in electrical conductivity. The major sources of noise are Johnson noise, thermal noise, generation–recombination (G–R) and 1/f noise [28,29,30,31]. The Johnson noise is due to random motion of charge carriers and does not significantly influence the performance of a highly biased bolometer. Thermal noise is due to thermal fluctuations in the sensing membrane and G–R noise is due to charge trapping. The 1/f noise (low frequency) is due to conductivity fluctuations and exhibits an inverse frequency dependence.

**Figure 8:**
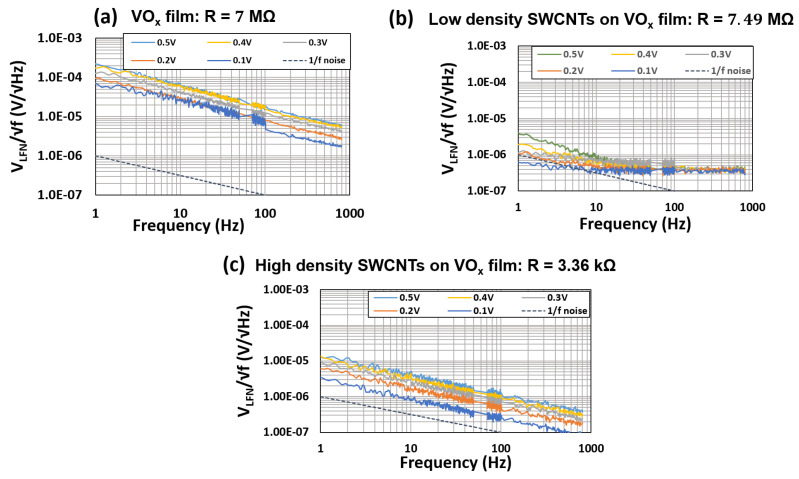
1/f noise amplitude spectral density as a function of bias voltage for devices made on: (**a**) the $VO_{x}$ film, (**b**) network with low SWCNT packing density on the $VO_{x}$ film and (**c**) network with high SWCNT packing density on the $VO_{x}$ film.

The spectral density of the 1/f noise signal ($V_{LFN}/\sqrt{f}$) was measured as a function of frequency and bias voltage for the devices made on the $VO_{x}$ films, devices with a low SWCNT packing density on the $VO_{x}$ film and devices with a high SWCNT packing density on the $VO_{x}$ film. As seen in the plots of Figure 8, the noise spectrum follows the 1/f noise for devices on the $VO_{x}$ film (Figure 8a) and in devices made with high packing density of SWCNTs dispersed on the $VO_{x}$ film surface (Figure 8c). The low packing density device shows 1/f noise only in the low frequency range of 1 to 10 Hz (Figure 8b). A significant reduction in the 1/f noise (about two orders of magnitude for low density dispersion) was measured following SWCNT dispersion. A moderate lowering (an order of magnitude) of the noise signal amplitude was also measured for the high packing density device.

**Figure 9:**
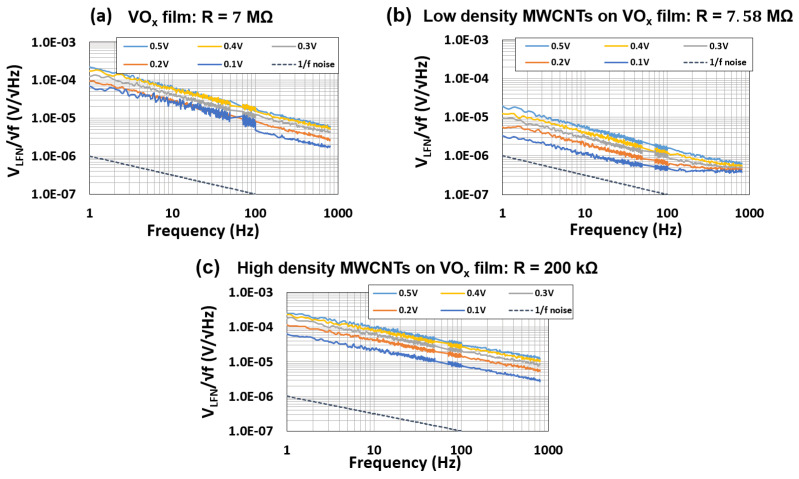
1/f noise amplitude spectral density as a function of bias voltage for devices made on: (**a**) the $VO_{x}$ film; (**b**) network with low MWCNT packing density on the $VO_{x}$ film and (**c**) network with high MWCNT packing density on the $VO_{x}$ film.

A similar trend was also observed for low and high packing densities of MWCNT dispersions on the $VO_{x}$ film (Figure 9b) [7].

No change in the noise signal amplitude was measured for the high packing density MWCNT devices. The resistances at room temperature, the noise signal amplitude at 0.1 V and 1 Hz and the TCR for the composite films are summarized in Table 1.

As seen in Table 1, the high packing density devices exhibit low resistance and low TCR values, factors that limit their performance in IR device applications. Notably, it is the low packing density SWCNT-based composite film devices that exhibit optimal properties in terms of the two orders of magnitude reduction in the noise signal. We see that at optimum CNT concentrations, it is possible to modify the chemical structure of the composite such that the amplitude of the noise signal is significantly reduced without any major influence on the conductivity or TCR of the $VO_{x}$ film. The orders of magnitude reduction in $V_{noise}$ while maintaining the TCR, coupled with a predicted increase in absorbance due to the CNTs, will result in a significant reduction in NETD (see Equation (2)).

Previous studies on $VO_{x}$-based bolometers have reported TCR values of −3.5% $K^{-1}$ [32], −2.3% ${}^{\circ }$C${}^{-1}$ [33], −1.88 to −2.85% ${}^{\circ }$C${}^{-1}$ [34] and −2.9% $K^{-1}$ [35]. The TCR values reported in this work (about −3.9% $K^{-1}$) are higher than the reported values and this is most likely associated with the higher resistivity of the $VO_{x}$ films under study in this work. The high resistivity of the $VO_{x}$ films also accounts for the higher noise reported in this study (on the order of $10^{-5}V/\sqrt{Hz}$, as shown in Figure 8a and Figure 9a and Table 1). Previous studies on $VO_{x}$ films have reported an order of magnitude lower 1/f noise in the range of $10^{-6}V/\sqrt{Hz}$ [36,37].

## 4. Conclusions

The results presented in this paper show that it is possible to modulate the electrical properties and noise signal amplitude of the $VO_{x}$–CNT composite by dispersing CNTs on a $VO_{x}$ film surface. The predicted chemical interactions between the 2D $VO_{x}$ film and the 1D CNTs result in electron transfer and creation of oxygen vacancies in the $VO_{x}$ film. The formation of such localized states in the band gap of the $VO_{x}$ reduces its energy band gap, resulting in an increased composite conductivity and a reduced TCR that is measured for a high density CNT dispersion on $VO_{x}$ film. Composites with low CNT concentrations—in the range of ≈0.30 μg/cm${}^{2}$—effectively combine the advantages of the $VO_{x}$ film (such as desirable R, high TCR and low 1/f noise) and the CNTs (unique 1D structures and promising electrical and optical properties). Though such composite films do not exhibit any significant reduction in TCR, the large reduction in the 1/f noise signal (especially for low density dispersion of SWCNT-based devices) makes them promising for applications as bolometer materials.

## Figures and Tables

**Table 1:** Comparison of device resistance, noise and TCR values obtained for devices fabricated on the $VO_{x}$ film, devices with low packing density of MWCNT and SWCNT on the $VO_{x}$ film, and devices with high MWCNT packing density on the $VO_{x}$ film.

Sample	Device *R* (Ω)	Noise at 0.1 V at 1 Hz ($V/\sqrt{\mathrm{Hz}}$)	TCR at 300 K (%/K)
VO${}_{x}$	7.0 M	$6\times 10^{-5}$	3.86
0.30 μg/cm${}^{2}$ MWCNT’s on VO${}_{x}$ film	7.5 M	$3\times 10^{-6}$	3.76
0.30 μg/cm${}^{2}$ SWCNT’s on VO${}_{x}$ film	7.4 M	$5\times 10^{-7}$	3.65
1.6 μg/cm${}^{2}$ MWCNT’s on VO${}_{x}$ film	200 K	$5\times 10^{-5}$	0.29
1.6 μg/cm${}^{2}$ SWCNT’s on VO${}_{x}$ film	3.86 K	$6\times 10^{-6}$	0.26

## Data Availability

Not applicable.

## Abbreviations

The following abbreviations are used in this manuscript:

$VO_{x}$	Vanadium oxide
TCR	Temperature coefficient of resistance
SWCNTs	Single-walled carbon nanotubes
MWCNTs	Multi-walled carbon nanotubes
VRH	Variable range hopping
1D	One-dimensional
2D	Two-dimensional
